# Relapsed/Progressive Disease and Its Prognostic Factors among Multiple Myeloma Patients Receiving Novel Agent Treatment in North East Peninsular Malaysia: A Single Centre Experience

**DOI:** 10.21315/mjms2020.27.5.7

**Published:** 2020-10-27

**Authors:** Hany Haqimi Wan Hanafi, Azlan Husin, Najib Majdi Yaacob, Abu Dzarr Abdullah

**Affiliations:** 1 Haematopoietic Stem Cell Transplant Unit, Hospital Universiti Sains Malaysia, Universiti Sains Malaysia, Kubang Kerian, Kelantan, Malaysia; 2 Department of Internal Medicine, School of Medical Sciences, Universiti Sains Malaysia, Kubang Kerian, Kelantan, Malaysia; 3 Unit of Biostatistics and Research Methodology, School of Medical Sciences, Universiti Sains Malaysia, Kubang Kerian, Kelantan, Malaysia

**Keywords:** multiple myeloma, relapsed, progressive, novel agent, Malaysia

## Abstract

**Background:**

Some multiple myeloma (MM) patients still relapse/progress despite novel agent therapy and relapse/progression in MM is therefore a vital area of ongoing research in the novel treatment era. This retrospective cohort study aimed to evaluate the time to relapse/progression (TTP) among MM patients who received novel agents and to determine the associated prognostic factors.

**Methods:**

This study included 89 MM patients treated at Hospital Universiti Sains Malaysia. We analysed the TTP and the type of relapse/progression (biochemical versus clinical), and a Cox proportional hazard model was used to identify the significant prognostic factors.

**Results:**

Sixty-four percent of patients had biochemical relapse/progression. The overall median TTP among MM patients who received the novel agent(s) was 29.33 months (95% CI: 21.36–37.29). The type of paraprotein at diagnosis (*P* = 0.026, *P =* 0.228), International Staging System (ISS) score (*P* = 0.036, *P* = 0.067) and autologous stem cell transplant (ASCT) (*P =* 0.002) were prognostic factors for relapse/progression by simple Cox regression, but ASCT was the only significant predictor detected by multiple Cox regression (*P* = 0.003).

**Conclusion:**

Our study reflects the importance of paraprotein monitoring to detect early features of relapse/progression. ASCT is the most prognostic factor that may lengthen the TTP.

## Introduction

Multiple myeloma (MM) is characterised by clonal proliferation of malignant plasma cells, which can result in an overabundance of monoclonal paraprotein (M-protein), leading to bony lesions, renal injury, hypercalcaemia, cytopenia and increased susceptibility to infections. MM accounts for 1% of all malignancies worldwide and 10% of all haematologic malignancies (1). The incidence rates of MM appear to be higher among males than females and higher in industrialised nations, such as North America, Australia, New Zealand and European countries than in Asian countries (2), although there is growing evidence that the incidence rates of MM are increasing in some Asian countries, such as in South Korea and Taiwan. However, there seem to be no unique clinical characteristics of MM that are peculiar to Asian patients (3). According to the National Cancer Registry, from 2007 to 2011, lymphoma and leukaemia were the fourth and sixth most common malignancies in Malaysia (4); however, data on MM was not reported, reflecting its rarity in the local population.

Before the era of novel agent therapy, MM was regarded as incurable and conventional combination chemotherapy led to a median survival of only three years or less. These combination chemotherapies included: i) vincristine, cyclophosphamide, melphalan and prednisone combination (VCMP); ii) vincristine, carmustine (BCNU), doxorubicin and prednisone combination (VBAP); and iii) vincristine, doxorubicin and prednisone combination (VAD) (5).

While there was some survival benefit, MM treatment has continued to advance. Two randomised trials conducted by Intergroup Français du Myeloma and the Medical Research Council revealed that high-dose therapy (HDT) supported with autologous stem cell transplantation (ASCT) improved the response rate, event-free survival and overall survival (OS) of patients with MM in comparison with conventional chemotherapy alone (6–7). Despite the significant impact of HDT plus ASCT on improving event-free survival, disease relapse and progression are still inevitable and this has led to a search for other strategies – hence, the discovery of novel agents (8).

There is still a lack of published information and data related to the pattern of the relapsed/progressive disease in Malaysia. The current published data on the survival of MM patients in the novel agent era is limited to western and developed countries.

In Malaysia, novel agents are costly and their availability is limited compared to conventional chemotherapy. Moreover, only the first and second generations of these novel agents are accessible. Our aim is therefore to study the occurrence of relapsed/progressive disease and to determine the median time to relapse/progression (TTP) and the influence of selected prognostic factors for relapse/progression among MM patients who received novel agent(s) therapy at a single Malaysian institution.

## Methods

This study was a retrospective cohort study involving a review of the medical records of MM patients at Hospital Universiti Sains Malaysia (HUSM) undergoing follow-up in the clinic or admission onto the ward; HUSM is a tertiary referral centre for haematological cases in Kelantan and Terengganu. Data was collected on MM patients based on records in the database registry from between 1 January 2006 and 30 April 2018, with an additional follow-up period of one year from 1 May 2018–30 April 2019. The total duration of this retrospective observation window was therefore 148 months.

This study only included patients who had been diagnosed with MM and had received induction treatment with the novel agent(s) at HUSM for at least four months. Patients with MM who had another primary cancer before MM, were missing a baseline evaluation of more than three variables or presented with primary plasma cell leukaemia were excluded from the study.

Two calculations for sample size were performed. To estimate the proportion of patients with biochemical and clinical relapse/progression, a standard formula for the estimation of a population’s proportion was used: *n* = (Z_α/Δ)^2 [P(1–P)], where *n* is the calculated sample size, Z_∝ is the critical value that corresponds to the level of confidence, Δ is the one-sided precision of estimate (margin of error) and P is the population’s proportion. A previous study reported that 85% of patients had asymptomatic (biochemical relapse) and 15% had clinical relapse (9). The sample size required for an estimation to a 95% confidence level and a 7.5% margin of error was therefore 88 patients.

To determine the prognostic factors for relapse/progression, the sample size was calculated again based on a comparison of two survival times using Power and Sample Size software (10). The largest sample size was calculated using the variable gender (female vs male) as the prognostic factor for relapse/progression. A previous study reported that the median survival time among females was 35 months (11). For a median survival time of 20 months for males to be significant at 5% type I error, 20% type II error, an accrual time of 132 months, an additional follow-up time of 12 months and a female-to-male ratio of 1, the required sample size calculated was 126 patients. Anticipating a 10% dropout due to missing data, the corrected sample size was 140 patients.

At HUSM, 170 MM patients were treated between 1 January 2006 and 30 April 2018. Of these 170 patients, only 89 fulfilled the eligibility criteria. We, therefore, did not apply a sampling method and all eligible patients were included in the study.

Data was entered into and analysed using IBM Statistical Package for the Social Sciences (SPSS) version 24. For categorical variables, the frequency of observations and percentages were calculated. The proportion of patients who were either asymptomatic or symptomatic was described by frequency and percentage.

Survival analysis was the statistical test of choice as the research objectives included time to an event, i.e. relapse/progression of MM. The outcome variable was TTP. Survival time referred to the months between the time of initiating the novel agent(s) and the TTP of the disease, which was identified based on biochemical features, with or without clinical features. Patients who relapsed/progressed were then classified as either asymptomatic (biochemical) or symptomatic (clinical). Due to the scarcity of patients who achieve complete remission (CR) or even stringent complete response (sCR), patients with very good partial response (VGPR) or partial response (PR) have been included in many studies and the term ‘relapsed/progressive disease’ is therefore collectively applied in many works in the literature. The censored observations included those patients who did not fulfil the definition of relapsed/progressive disease, patients with early death or non-myeloma-related death and patients who had defaulted follow-up.

Median progression time for all categorical independent variables was estimated using Kaplan-Meier analysis. The median survival time (in months) and its 95% confidence interval (CI) were calculated. Both univariable (simple) and multivariable (multiple) Cox proportional hazard regression analyses were performed to identify the important predictors of relapsed/progressive disease. Simple Cox regression analysis was conducted to provide a preliminary idea of the potential prognostic importance at univariable level using the Enter method. Variables with a *P*-value of ≤ 0.25 were included in the variable selection for the multiple Cox regression analysis. Variables with a *P*-value of > 0.25, but which were clinically important or biologically sound, were also included in the variable selection. Variable selection was conducted using both the forward and backward likelihood ratio methods to obtain the preliminary main effect model, which was confirmed using the Enter method to obtain the preliminary final model. The hazard function plot and the log-minus-log plot were used to examine the assumption of proportional hazard. The final model is presented as crude and adjusted hazard ratio (HR), 95% CI, the value of the Wald statistic and the *P*-value. The level of significance α was set at 0.05 (two-tailed).

## Results

Out of 170 patients, only 89 who received novel agent(s) treatment were recruited into the study (Table 1). At the end of the study, 50 (56.2%) MM patients fulfilled the criteria for relapsed/progressive disease and the remaining 39 (43.8%) patients were censored. Seventy-five (84.3%) were aged 65 years or less; 50 (56.2%) were male and 39 (43.8%) female; and 29 (29.2%) had renal insufficiency at diagnosis, while 11 (12.4%) had cardiovascular disease and 8 (9.0%) had chronic respiratory disease.

The disease and treatment characteristics of the MM patients are shown in Table 2. The majority of patients (82.0%) had an unknown baseline cytogenetic profile at diagnosis. Fourteen (87.5%) were in the standard risk category, while only 2 (12.5%) were in the high-risk group. Most of the recruited patients had IgG paraprotein at diagnosis (60, 68.2%) followed by IgA (19, 21.6%) and free light chain (FLC)-only paraprotein (9, 10.2%); only 1 patient was without a known type of paraprotein at diagnosis. Of the 89 patients recruited, 54 had a baseline Kappa/Lambda FLC ratio at diagnosis, and the majority of those (46, 85.2%) had an abnormal Kappa/Lambda ratio.

Sufficient data for risk stratification using the ISS at diagnosis was only available for 71 patients. Most of these had advanced ISS scores of III (44, 62.0%), followed by scores of II (19, 26.8%) and I (8, 11.3%). The proportion of patients with normal lactate dehydrogenase (LDH) at diagnosis was higher (64, 71.9%) than those with abnormal LDH (25, 28.1%).

We advocate for the use of bortezomib (Velcade^®^) as a proteasome inhibitor (PI) and thalidomide (Thalomid^®^) or lenalidomide (Revlimid^®^) as immunomodulatory drugs (IMiD). PI-only treatment consists of bortezomib plus dexamethasone (Vel-dex), while IMiD-only treatment consists of either thalidomide plus dexamethasone (Thal-dex) or lenalidomide plus dexamethasone (Vel-dex). The PI plus IMiD combination comprises either bortezomib, thalidomide and dexamethasone (VTd) or bortezomib, lenalidomide and dexamethasone (VRd). A total of 52 (58.4%) patients were on a combination of PI and IMiD during induction treatment, while the numbers of patients on IMiD-only and PI-only treatment were 26 (29.2%) and 11 (12.4%), respectively.

Of the recruited patients, 41 (46.1%) underwent ASCT at some point during the study period. Only 7 (8.0%) subjects achieved sCR, while 38 (43.2%) achieved VGPR, 20 (22.7%) achieved CR and 23 (26.1%) achieved PR. Notably, one patient had an unknown response due to early death during treatment and was therefore considered missing data. Of the 50 relapsed/progressive MM patients, 32 had asymptomatic (biochemical) relapsed/progressive disease, and the remaining 18 patients had demonstrated features of symptomatic (clinical) relapsed/progressive disease (Table 3).

**Figure 1:**
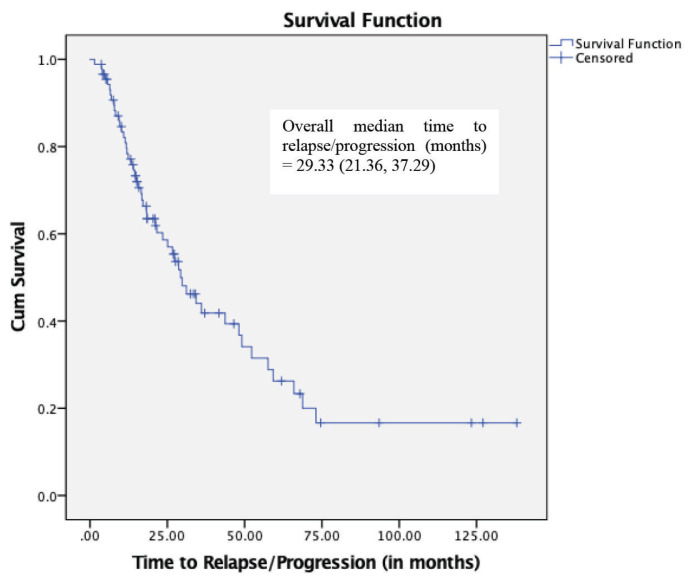
Kaplan-Meier survival curve for TTP in multiple myeloma patients receiving novel agent therapy (*n* = 89)

**Figure 2:**
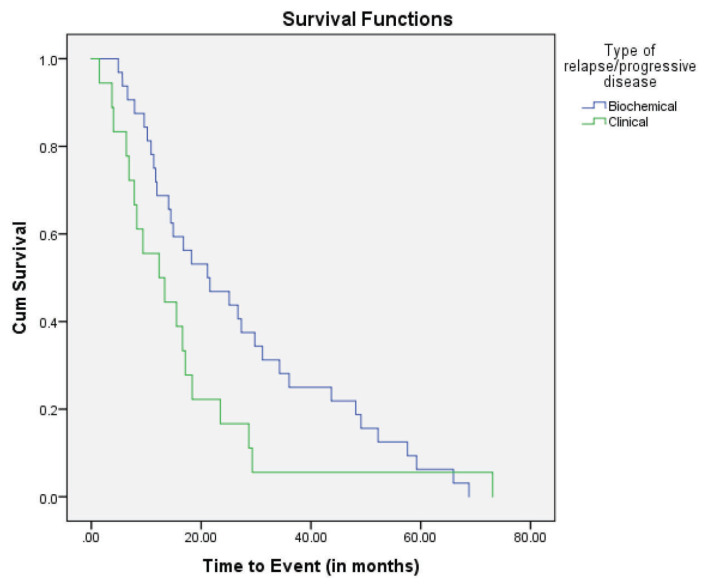
Kaplan-Meier survival curve for TTP for type of relapse/progression (*n* = 89)

The overall median TTP (months) was 29.33 (95% CI: 21.36–37.29) (Figure 1). In terms of the type of relapse/progression, patients with asymptomatic (biochemical) type exhibited a longer TTP than those with symptomatic (clinical) type. The median TTP (months) for the asymptomatic patients was 21.17 (95% CI: 9.60–32.75), while the median TTP for the symptomatic patients was 12.36 (95% CI: 4.16–20.56) (Figure 2).

**Figure 3:**
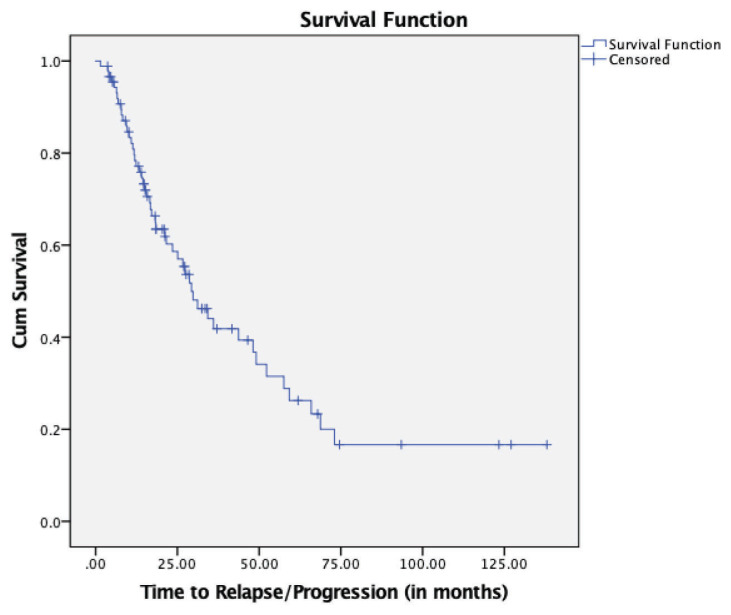
Kaplan-Meier survival curve for TTP for paraprotein at diagnosis (*P* = 0.021)

**Figure 4:**
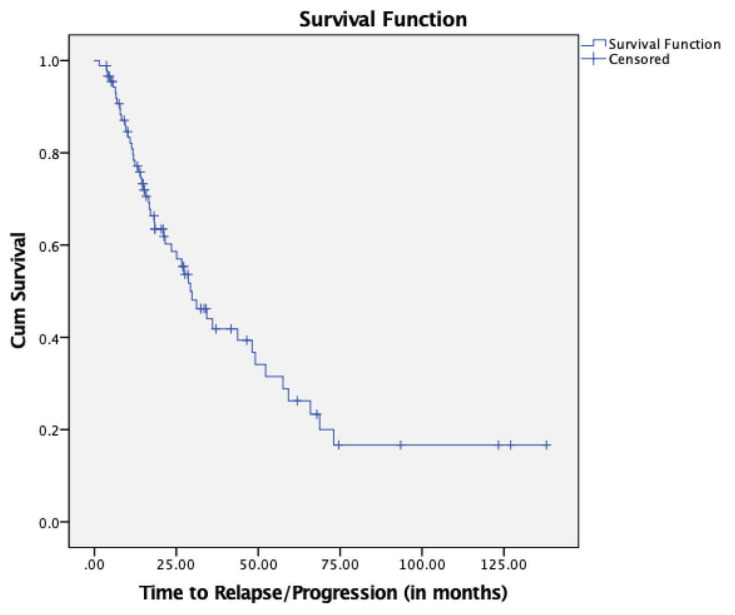
Kaplan-Meier survival curve for TTP for ASCT (*P* = 0.001)

**Figure 5:**
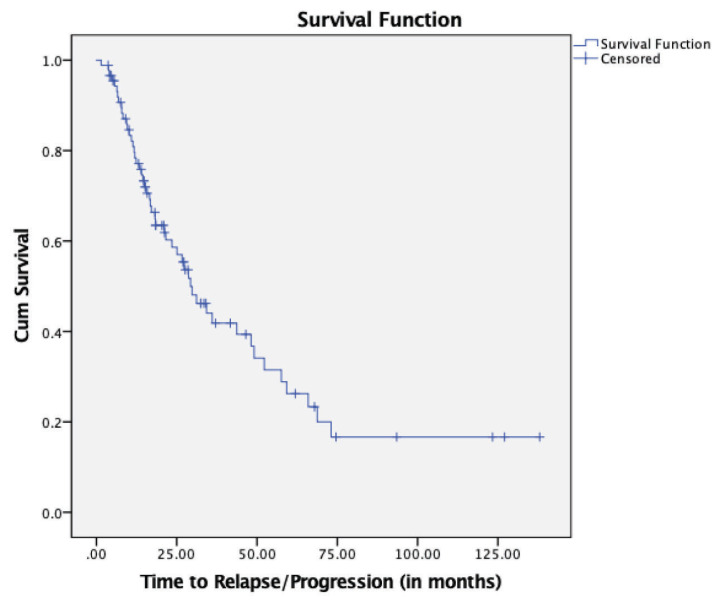
Kaplan-Meier survival curve for TTP for best response achieved (*P* < 0.001)

To further examine the effect of the novel agent(s) across various risk groups, we analysed the differences in TTP for known adverse factors. Patients with IgG paraprotein at diagnosis had prolonged median TTP compared to IgA and FLC-only paraproteins (*P* = 0.021) (Figure 3). Moreover, the patients who underwent ASCT had a significantly longer TTP than non-ASCT patients (48.16 months versus 18.38 months, *P* = 0.001) (Figure 4). Lastly, the patients who achieved sCR had the best survival outcomes, followed, in order, by those who achieved CR, VGPR and PR (*P* < 0.001) (Figure 5).

Various potential prognostic factors were evaluated using simple Cox proportional hazard regression to identify possible significant independent prognostic factors for relapse/progression in MM patients receiving novel agent(s) therapy. There were only three such unadjusted factors, as shown in Table 4: type of paraprotein at diagnosis (*P* = 0.026, *P* = 0.228), ISS score (*P* = 0.036, *P* = 0.067) and ASCT (*P* = 0.002).

The three variables with a *P*-value below 0.25 in the simple Cox regression analysis (paraprotein at diagnosis, ISS and ASCT) and variables that are clinically important (age and best response achieved) were then included in the multivariable analysis. Eventually, only ASCT (*P* = 0.003) was found to be an independent prognostic factor that could influence the risk of relapse/progression in MM patients (Table 5). The hazard ratio for relapse/progression for patients who did not undergo ASCT was 2.72 (95% CI: 1.40–5.29; *P* = 0.003). At any particular time, patients who did not undergo ASCT had a 2.7 times higher risk of relapse/progression, and we are 95% confident that the real value lies between 1.4 times and 5.3 times.

## Discussion

To the best of our knowledge, at the time of writing, there are no published studies on the relapse/progression of MM in Malaysia, particularly for the Malay race, who are the majority of our research population. Most previous studies have involved Caucasians and East Asians.

Prolongation of OS is traditionally accepted as the gold standard for demonstrating the clinical benefit of a particular therapy in malignancy. Nevertheless, the use of OS as the primary endpoint in clinical trials might be troublesome because of the need for large numbers of patients and prolonged follow-up and the confounding effects of crossover or possible post-progression therapies (12). Some randomised clinical trials of novel treatment were designed with OS as the primary endpoint, but, notably, some used TTP or progression-free survival (PFS) as the primary endpoint.

This study found an overall median TTP of 29.33 months (95% CI: 21.36–37.29). In the VISTA trial, the median TTP among newly diagnosed and untreated patients receiving bortezomib plus melphalan and prednisolone was 24 months, which is comparable with our findings (13–14). However, there is a scarcity of previous data to compare our median TTP with those not treated with a novel agent, since OS has been the primary endpoint in many clinical trials. There is also no similar data for TTP among Chinese populations, but results from a multicentre analysis of treatment outcomes in newly diagnosed Chinese patients revealed a median OS of 54 months and a PFS of 26 months (15). Data on survival from India and other parts of the Asian region remains scarce.

The majority of our patients at first relapse/progression had an asymptomatic (biochemical) pattern and this reflects the importance of paraprotein monitoring at timely and regular intervals, consistent with previous literature (9). In our centre, this is tested at diagnosis and every two months during the course of treatment and follow-up.

Of the 50 relapsed/progressive patients in our study, 18 had clinical relapse without evidence of prior biochemical relapse. These clinical relapses occurred earlier, at a median TTP of 12.36 months, than those with biochemical relapse, at 21.17 months. The occurrence of the clinical features of relapse preceding the elevation of serum paraprotein or involving FLC is questionable, since plasma cell malignancy is a spectrum of disease, from monoclonal gammopathy of undetermined significance (MGUS) to smouldering myeloma, clinical MM and, rarely, plasma cell leukaemia. In addition, MM patients may exhibit different disease phases involving multiple periods of response and remission. It may be that the biochemical parameters of our MM patients were not adequately monitored to some degree, especially during the initial establishment of our centre, and a post-hoc analysis looking at the prognostic factors of those with biochemical and clinical relapse would increase the value of this study.

Many identified prognostic factors, such as staging, could influence the risk of relapse/progression in MM. Staging in MM began with the Durie–Salmon staging system in 1975, which incorporated levels of paraprotein, the number of lytic bone lesions, haemoglobin levels, serum calcium levels and creatine levels (16). This was followed by the much simpler, but robust, ISS in 2005, based on β_2_-microglobulin and serum albumin levels, which became the standard risk stratification for myeloma patients (17). In 2015, the Revised International Staging System (R-ISS) became the new standard risk stratification algorithm, with an improved prognostic power and combined the chromosomal abnormalities detected by fluorescence in situ hybridisation (FISH) with serum LDH (18). The R-ISS has since been validated as the best prognostic system and can identify three different groups of patients with clearly different outcomes (19).

Our study used the ISS as the staging system variable because FISH is costly and not routinely performed. From our simple Cox regression analysis, the ISS was significant as an unadjusted prognostic factor, but no longer significant when evaluated with the other variables included in the multiple Cox regression. However, the ISS remains an important prognostication tool, particularly in patients who were treated upfront with novel agent–based therapy (20).

Measurement of the Kappa/Lambda FLC ratio and serum β_2_-microglobulin and the determination of cytogenetic profiles by FISH was not widely available in our centre before 2010 due to the cost, and this missing data might hinder the use of the exact values of these variables in the pattern of relapse/progression in our patients. Kappa/Lambda FLC ratio in newly diagnosed MM patients might be of baseline prognostic value, but serial measurements might not provide added value (21).

In the simple Cox regression analysis, the TTP was significantly correlated with the type of paraprotein at diagnosis, ISS score and ASCT, but the analysis did not offer sufficiently dependable information. The use of the multiple Cox regression analysis optimised and simplified the combination of those variables, leading to the conclusion that ASCT was the only independent prognostic factor in determining the risk of relapse/progression in MM patients receiving novel agent(s) therapy. Patients who did not undergo ASCT were 2.72 times more likely to relapse/progress than those who did.

The prognostic value of ASCT is well known and this finding is consistent with the majority of published data. Before the era of novel agents, HDT followed by ASCT demonstrated better survival rates than conventional chemotherapy alone, whether it was performed as upfront or as rescue treatment (22). A systematic review and meta-analysis by Dhakal et al. (23) demonstrated that HDT/ASCT in the novel agent era was associated with superior PFS and had a comparable safety profile and higher CR rates than standard therapy alone, i.e., induction with the novel agent.

In comparison with previous studies, the introduction of novel agents has significantly increased the survival of MM patients but with different significant prognostic parameters. Kastritis et al. (20), for example, found that survival improvement is mainly evident in younger patients and patients with a lower ISS stage.

A previous study investigating OS by paraprotein class found that myeloma patients with IgG had the longest overall survival (median: 2.5 years; 95% CI: 2.3–2.7), followed by IgA (median: 2.3 years; 95% CI: 2.1–2.6) and light chain–only paraprotein (median: 1.9 years; 95% CI: 1.5–2.3) (24). Similarly, our patients with IgG paraprotein at diagnosis had a longer median TTP than those with IgA paraprotein. However, observations for FLC-only paraprotein cannot be made, mainly due to the very small proportion of patients with FLC-only paraprotein in this study.

During the earlier phrase of incorporating novel agents for treating our myeloma patients, the choice of novel agent(s) was hugely influenced by cost. With the subsequent evolving guidelines and evidence, many efforts have been made to increase the usage of novel agent(s) and to follow transplant eligibility criteria. However, our study did not thoroughly evaluate the association between the various types of novel agent(s) chosen at induction and the risk of developing subsequent relapse/progression.

The pharmacoeconomic aspect of newer drugs in cancer therapy, including MM, has been a subject of interest in recent years. It has been suggested that using less expensive regimes early in the disease course could be more cost-effective because the duration of the therapy might decrease with each relapse. Moreover, the cost of regimes may fall further with the emergence of generics or other competitors with the same mode of action, which could create downward price pressures (25). Even though there has been a marked increase in healthcare costs—attributable to improved treatments for MM and changes in disease management—the mortality rate in patients with MM has been reduced significantly, reflecting the survival benefit of the novel agent(s) (26).

This study has several limitations. Firstly, the design of the study itself—a retrospective cohort study—relies on secondary data from medical records and is therefore subject to mis-documentation and reporting bias, and we dealt with several missing data items, including baseline cytogenetic profile, Kappa/Lambda ratio and serum β_2_-microglobulin. Secondly, our survival analysis involved determining TTP as the primary endpoint. OS is the most reliable endpoint in clinical studies involving malignancy, but it requires more time than both TTP and PFS, which can overcome some of the limitations of OS, i.e. they are not affected by subsequent therapies and the follow-up periods required are shorter. Thirdly, there were only 170 patients with MM in our centre during the study period, and fewer than half received novel agent(s). As the number of patients included is lower than the required sample size, the power of this study is therefore less than 80% (calculated post-hoc power is 66.4%). It is therefore recommended that, in the future, a similar study should be conducted involving multiple centres from all over the country with a higher number of participants.

The strength of this study lies in the inclusion criteria, which were quite broad and with limited exclusion criteria and which produced a study population that was more representative of the target population, with results that were, therefore, more generalisable. The results of this study offer beneficial information regarding MM patients in Malaysia that may help in providing effective patient care and improving the survival rate.

## Conclusion

In conclusion, the majority of patients with relapsed/progressive MM initially tend to exhibit biochemical relapse more than clinical relapse, and this reflects the importance of paraprotein monitoring at regular intervals. The overall median TTP among MM patients receiving novel agent(s) therapy was 29.33 months, and this figure is comparable to previous studies in the literature. ASCT was the only prognostic factor that might prolong the TTP in MM patients, in addition to novel agent(s) as the induction therapy. This study did not provide enough evidence to show the one novel agent is superior to another agent or combination.

## Figures and Tables

**Table 1:** Sociodemographic baseline characteristics of multiple myeloma patients receiving novel agent (*n* = 89)

Baseline characteristic	Frequency *n* (%)	Relapsed/Progressive disease	
		Yes, *n* (%)	Censored, *n* (%)
Age at diagnosis (years)			
≤ 65	75 (84.3%)	44 (58.7%)	31 (41.3%)
> 65	14 (15.7%)	6 (42.9%)	8 (57.1%)
Sex, *n* (%)			
Male	50 (56.2%)	29 (58.0%)	21 (42.0%)
Female	39 (43.8%)	21 (53.8%)	18 (46.2%)
Comorbidities at diagnosis, *n* (%)			
Renal insufficiency			
Yes	26 (29.2%)	13 (50.0%)	13 (50.0%)
No	63 (70.8%)	37 (58.7%)	20 (41.3%)
Cardiovascular disease			
Yes	11 (12.4%)	5 (10.0%)	6 (15.4%)
No	78 (87.6%)	45 (90.0%)	33 (84.6%)
Chronic respiratory disease			
Yes	8 (9.0%)	5 (62.5%)	3 (37.5%)
No	81 (91.0%)	45 (55.6%)	36 (44.4%)

**Table 2:** Disease parameter and treatment characteristics of multiple myeloma patients receiving novel agent (*n* = 89)

Baseline characteristic	Frequency *n* (%)	Relapsed/Progressive disease	
		Yes, *n* (%)	Censored, *n* (%)
Cytogenetic risk^a^			
Standard	14 (87.5%)	7 (50.0%)	7 (50.0%)
High	2 (12.5%)	1 (50.0%)	1 (50.0%)
Paraprotein^b^			
IgG	60 (68.2%)	33 (55.0%)	27 (45.0%)
IgA	19 (21.6%)	14 (73.7%)	5 (26.3%)
FLC-only	9 (10.2%)	2 (22.2%)	7 (77.8%)
Kappa/Lambda ratio^c^			
Normal	8 (14.8%)	3 (37.5%)	5 (62.5%)
Abnormal	46 (85.2%)	21 (45.7%)	25 (54.3%)
International Staging System^d^			
I	8 (11.3%)	3 (37.5%)	5 (62.5%)
II	19 (26.8%)	13 (68.4%)	6 (31.6%)
III	44 (62.0%)	27 (61.4%)	17 (38.6%)
International Staging System^d^			
I	8 (11.3%)	3 (37.5%)	5 (62.5%)
II	19 (26.8%)	13 (68.4%)	6 (31.6%)
III	44 (62.0%)	27 (61.4%)	17 (38.6%)
LDH^f^ at diagnosis (U/L)			
< 480	64 (71.9%)	35 (54.7%)	29 (45.3%)
≥ 480	25 (28.1%)	15 (60.0%)	10 (40.0%)
Type of induction treatment			
PI only	11 (12.4%)	5 (45.5%)	6 (54.5%)
IMiDs only	26 (29.2%)	19 (73.1%)	7 (26.9%)
PI + IMiDs	52 (58.4%)	26 (50.0%)	26 (50.0%)
Autologous stem cell transplant			
Yes	41 (46.1%)	22 (53.7%)	19 (46.3%)
No	48 (53.9%)	28 (58.3%	20 (41.7%)
Best response achieved (IMWG)^e^			
sCR	7 (8.0%)	3 (42.9%)	4 (57.1%)
CR	20 (22.7%)	7 (35.0%)	13 (65.0%)
VGPR	38 (43.2%)	24 (63.2%)	14 (36.8%)
PR	23 (26.1%)	16 (69.6%)	7 (30.4%)

Notes:

a Missing data 82.0% (*n* = 16);

b Missing data 1.1% (*n* = 88);

c Missing data 39.3% (*n* = 54);

d Missing data 20.2% (*n* = 71);

e Missing data 1.1% (*n* = 88);

f Normal LDH level in HUSM lab < 480 U/L

**Table 3:** Types of relapsed/progressive disease with baseline characteristics (*n* = 50)

Baseline characteristics	Asymptomatic (biochemical) *n* (%) = 32 (64.0%)	Symptomatic (clinical) *n* (%) = 18 (36.0%)
Age at diagnosis (years)		
≤ 65	28 (87.5%)	16 (88.9%)
> 65	4 (12.5%)	2 (11.1%)
Sex		
Male	22 (68.8%	7 (38.9%)
Female	10 (31.3%)	11 (61.1%)
Cytogenetic risk		
Standard	6 (18.8%)	1 (5.6%)
High	0 (0.0%)	1 (5.6%)
Unknown	26 (81.2%)	16 (88.8%)
Paraprotein		
IgG	22 (68.8%)	11 (61.1%)
IgA	9 (28.1%)	5 (27.8%)
FLC-only	0 (0.0%)	2 (11.1%)
Unknown	1 (3.1%)	0 (0.0%
Kappa/Lambda ratio		
Normal	0 (0.0%)	3 (16.7%)
Abnormal	16 (50.0%)	5 (27.8%)
Unknown	16 (50.0%)	10 (55.5%)
International Staging System		
I	1 (3.1%)	2 (11.1%)
II	9 (28.1%)	4 (22.2%)
III	18 (56.3%)	9 (50.0%)
Unknown	4 (12.5%)	3 (16.7%)
LDH at diagnosis (U/L)		
< 480	22 (68.8%)	13 (72.2%)
≥ 480	10 (31.3%)	5 (27.8%)
Comorbidities at diagnosis		
Renal insufficiency		
Yes	8 (25.0%)	5 (27.8%)
No	24 (75.0%)	13 (72.2%)
Cardiovascular disease		
Yes	2 (6.3%)	3 (16.7%)
No	30 (93.8%)	15 (83.3%)
Chronic respiratory disease		
Yes	4 (12.5%)	1 (5.6%)
No	28 (87.5%)	17 (94.4%)
Type of induction treatment		
PI only	3 (9.4%)	2 (11.1%)
IMiDs only	11 (34.4%)	8 (44.4%)
PI + IMiDs	18 (56.3%)	8 (44.4%)
Best response achieved (IMWG)		
sCR	2 (6.3%)	1 (5.6%)
CR	3 (9.4%)	4 (22.2%)
VGPR	20 (62.5)	4 (22.2%)
PR	7 (21.9%)	9 (50.0%)

**Table 4:** Prognostic factors for relapsed/progressive disease among multiple myeloma patients receiving novel agent using simple Cox regression (*n* = 89)

Variable	Frequency *n* (%)	Crude HR^a^ (95% CI^b^)	Wald statistics	*P-*value^c^
Age at diagnosis (years)				
≤ 65	75 (84.3%)	1.00		
> 65	14 (15.7%)	0.97 (0.41, 2.29)	0.01	0.939
Sex				
Male	50 (56.2%)	1.00		
Female	39 (43.8%)	0.91 (0.52, 1.60)	0.11	0.739
Cytogenetic risk				
Standard	14 (87.5%)	1.00		
High	2 (12.5%)	1.05 (0.12, 9.03)	0.00	0.965
Paraprotein				
IgG	60 (68.2%)	1.00		
IgA	19 (21.6%)	2.06 (1.09, 3.89)	4.94	0.026
FLC-only	9 (10.2%)	0.42 (0.10, 1.73)	1.45	0.278
Kappa/Lambda ratio				
Normal	8 (14.8%)	1.00		
Abnormal	46 (85.2%)	1.10 (0.33, 3.71)	0.02	0.880
International Staging System				
I	8 (11.3%)	1.00		
II	19 (26.8%)	3.91 (1.09, 13.98)	4.39	0.036
III	44 (62.0%)	3.07 (0.93, 10.16)	3.36	0.067
LDH at diagnosis (U/L)				
< 480	64 (71.9%)	1.00		
≥ 480	25 (28.1%)	1.26 (0.68, 2.32)	0.55	0.459
Comorbidities at diagnosis				
Renal insufficiency				
No	63 (70.8%)	1.00		
Yes	26 (29.2%)	0.94 (0.50, 1.77)	0.04	0.843
Cardiovascular disease				
No	78 (87.6%)	1.00		
Yes	11 (12.4%)	1.18 (0.47, 2.99)	0.12	0.725
Chronic respiratory disease				
No	81 (91.0%)	1.00		
Yes	8 (9.0%)	0.61 (0.24, 1.54)	1.11	0.292
Type of induction treatment				
PI only	11 (12.4%)	1.00		
IMiDs only	26 (29.2%)	1.36 (0.50, 3.67)	0.37	0.546
PI + IMiDs	52 (58.4%)	1.12 (0.43, 2.93)	0.05	0.817
Autologous stem cell transplant				
Yes	41 (46.1%)	1.00		
No	48 (53.9%)	2.69 (1.46, 4.96)	10.06	0.002
Best response achieved (IMWG)				
sCR	7 (8.0%)	1.00		
CR	20 (22.7%)	1.34 (0.34, 5.27)	0.18	0.674
VGPR	38 (43.2%)	3.29 (0.97, 11.12)	3.66	0.056
PR	23 (26.1%)	7.20 (2.00, 25.93)	9.13	0.003

Notes:–

a Hazard ratio;

b Confidence interval;

c Wald test applied; level of significance was set at < 0.25

**Table 5:** Prognostic factors for relapsed/progressive disease among multiple myeloma patients receiving novel agent using multiple Cox regression

Variable	Frequency *n* (%)	Adjusted HR (95% CI)	Wald statistic	*P-*value
Autologous stem cell transplant				
Yes	41 (46.1%)	1.00		
No	48 (53.9%)	2.72 (1.40, 5.29)	8.68	0.003

Notes: Level of significance was set at < 0.05; Adj. b = Adjusted regression coefficient; Adj. HR = Adjusted hazard ratio; Forward, and Backward LR stepwise method was applied; Proportional hazard assumption by hazard function plot and log-minus-log plot were checked and assumptions were met
